# Immune and Hereditary Thrombotic Thrombocytopenic Purpura: Can ADAMTS13 Deficiency Alone Explain the Different Clinical Phenotypes?

**DOI:** 10.3390/jcm12093111

**Published:** 2023-04-25

**Authors:** Stefano Lancellotti, Monica Sacco, Maira Tardugno, Antonietta Ferretti, Raimondo De Cristofaro

**Affiliations:** 1 Servizio Malattie Emorragiche e Trombotiche, Fondazione Policlinico Universitario “A. Gemelli” IRCCS, 00168 Roma, Italy; stefano.lancellotti@policlinicogemelli.it; 2 Dipartimento di Medicina e Chirurgia Traslazionale, Facoltà di Medicina e Chirurgia “Agostino Gemelli”, Università Cattolica S. Cuore, 00168 Roma, Italy; monicasacco.89@gmail.com (M.S.); maira.tardugno@gmail.com (M.T.);

**Keywords:** pathophysiology of iTTP, autoantibodies to ADAMTS-13, thrombotic microangiopathy

## Abstract

Thrombotic thrombocytopenic purpura (TTP) is a thrombotic microangiopathy caused by a hereditary or immune-mediated deficiency of the enzyme ADAMTS13 (a disintegrin and metalloproteinase with a thrombospondin type 1 motif, member 13). TTPs are caused by the following pathophysiological mechanisms: (1) the presence of inhibitory autoantibodies against ADAMTS13; and (2) hereditary mutations of the *ADAMTS13* gene, which is present on chromosome 9. In both syndromes, TTP results from a severe deficiency of ADAMTS13, which is responsible for the impaired proteolytic processing of high-molecular-weight von Willebrand factor (HMW-VWF) multimers, which avidly interact with platelets and subendothelial collagen and promote tissue and multiorgan ischemia. Although the acute presentation of the occurring symptoms in acquired and hereditary TTPs is similar (microangiopathic hemolytic anemia, thrombocytopenia, and variable ischemic end-organ injury), their intensity, incidence, and precipitating factors are different, although, in both forms, a severe ADAMTS13 deficiency characterizes their physiopathology. This review is aimed at exploring the possible factors responsible for the different clinical and pathological features occurring in hereditary and immune-mediated TTPs.

## 1. Introduction

Thrombotic microangiopathies (TMAs) are a heterogeneous group of syndromes associated with the generation of disseminated microthrombi responsible for a clinical triad composed of microangiopathic hemolytic anemia (MAHA), thrombocytopenia, and variable ischemic end-organ injury [1]. Such syndromes, although stemming from different pathophysiological mechanisms, present with a similar clinical phenotype. The main diagnostic aspects of different TMAs are summarized in Table 1, which includes the most recent clinical form associated with coronavirus disease-19 (COVID-19), whose pathogenesis is still under investigation [2]. The latter, although it would resemble the pathophysiology of complement-mediated TMAs, shows genetic and functional evidence of complement dysregulation. Thrombotic thrombocytopenic purpura (TTP) is caused by a hereditary (cTTP) or immune-mediated (iTTP) deficiency of the enzyme ADAMTS13 (a disintegrin and metalloproteinase with a thrombospondin type 1 motif, member 13) [3,4] and is one of the best characterized TMAs [5,6,7,8]. After the initial discovery of the main culprit for TTPs, further understanding of the underlying pathophysiology of TTP has led to significant advancements in the diagnosis [9,10,11,12] and clinical management of these patients [13,14,15], as well as increasing interest in issues related to TTP survivorship. Although the acute clinical phenomena occurring in iTTP and cTTP are similar (MAHA, thrombocytopenia, and variable ischemic end-organ injury), their intensity, incidence, and precipitating factors are different, despite the fact that in both forms, a severe ADAMTS13 deficiency characterizes their physiopathology.

The present review is aimed at exploring the possible factors responsible for the different clinical and pathological features of TTP.

## 2. TTP Pathophysiology

TTPs are caused by different pathophysiological mechanisms, which include the following: (1) the presence of inhibitory autoantibodies against ADAMTS13; and (2) hereditary mutations of the *ADAMTS13* gene, which is present on chromosome 9q34 [4]. In both syndromes, TTP results from a severe deficiency of ADAMTS13, which is responsible for the impaired proteolytic processing of high-molecular-weight von Willebrand factor (HMW-VWF) multimers that, under shear stress of >30 dyn/cm^2^, are stretched and form long strings that are able to avidly interact with platelets and subendothelial collagen. Moreover, the longer the VWF multimer, the higher its sensitivity to shear stress [16]. In fact, the tensile force F(j) to the inside of any sphere pair j in a chain with N dimers, such as in a VWF multimer, is the sum of the forces on all the outer dimer pairs. The total tensile force, F(j), is calculated as follows [17]:

(1) $$F(j)\approx \sum_{i=j}^{N}f[ix(d+2a)]\approx \frac{\left(N+j\right)\left(N+1-j\right)}{2}f(d+2a)$$

where f(i) is the normal force between two spheres that are a certain distance (x) apart. From Equation (1), a is the radius of the sphere; the normal force on a monomer in the center of a multimer is approximately proportional to N^2^ (i.e., when j = 1), whereas the force on a monomer at the end of a multimer is proportional to N (i.e., when j = N). This is why the prothrombotic potential of VWF multimers occurs in the microcirculation. Here, hemodynamic principles indicate the presence of the highest shear stress in the entire circulatory tree that can induce a drastic conformational change and stretch the VWF multimers that interact and aggregate a great number of blood platelets with the resulting ischemic effects.

## 3. Immune TTP (iTTP)

Most patients with iTTP have detectable anti-ADAMTS13 autoantibodies that may have inhibitory or non-inhibitory features [18,19,20,21]. The former block the proteolysis of HMW-VWF, whereas ADAMTS13 clearance from the circulatory tree is accelerated by non-inhibitory antibodies [18]. The latter were found to target several domains of ADAMTS13. The spacer domain of the metalloprotease represents a hotspot for interacting antibodies. In fact, antibodies against the spacer domain are present in the majority of iTTP patients, inhibiting the enzyme’s activity [22,23,24,25]. The mechanisms of action of non-inhibitory antibodies, also defined as “clearing” antibodies, are not fully clarified. For instance, Thomas M.R. and colleagues found non-inhibitory antibodies in 15 out of 43 patients during an acute iTTP episode [21]. Moreover, the ADAMTS13 antigen levels were found to be very low in the early phase of acute iTTP, and the patients falling in the lowest quartile of the ADAMTS13 antigen level showed the highest mortality rate [21]. ADAMTS13, which would usually circulate in a “closed” globular conformation, was found in an “open” conformation, both during acute iTTP episodes and phases of clinical remission with subnormal ADAMTS13 levels [26]. In studies by Roose and colleagues, autoantibodies against ADAMTS13 have been described to induce a conformational transition of the ADAMTS13 molecules from a native “closed” state to an “open” state [11,26], causing the exposure of cryptic epitopes in the spacer region. Furthermore, different autoantibodies directed against the distal carboxy terminal of ADAMTS13, where CUB domains are present, modulate its susceptibility to inhibitory antibodies [27]. The complete list of the negative activities exerted by these autoantibodies in the pathophysiology of iTTP is yet to be fully characterized. The detailed and specific mechanisms leading to the loss of tolerance for ADAMTS13 in iTTP are still far from being identified. Similar to any autoimmune disorder, environmental factors such as female sex, ethnicity/race, or obesity may represent risk factors for iTTP [28,29,30,31]. Some human leukocyte antigen (HLA) haplotypes seem to be associated with iTTP occurrence. A higher prevalence of Class II locus DRB1*11 and DQB1*03 alleles was found in Caucasian patients. At variance, the HLA-DRB1*04 haplotype showed a protective effect in this population [32,33,34,35], while in African patients, the frequency of this haplotype is markedly reduced. This haplotype pattern could explain the 8-fold higher incidence of iTTP among black people in the United States [29,30]. In another study, the HLA-DRB1*11 or HLADRB1*04 alleles did not show a protective or predisposing effect on iTTP in a cohort of Japanese patients [36]. By contrast, HLA-DRB1*08:03, HLA-DRB3/4/5*blank, HLA-DQA1*01:03, and HLA-DQB*06:01 have been suggested as possible risk factors [36]. ADAMTS13 deficiency is a necessary but not always sufficient element to trigger an iTTP relapse [37,38,39]. Hence, it is likely that other synergic mechanisms may provide a “second hit”, responsible for iTTP initiation [40]. In this respect, the activation of the alternative pathway of the complement system may act as a facilitating mechanism to induce an acute phase of iTTP [41,42,43], where ULVWF multimers can be involved in the activation of the alternative complement pathway [44]. It has to be noted that the complement factors, VWF multimers, and the ADAMTS13 level of patients in remission of iTTP were shown to be entangled [42]. High levels of HMW multimers were in fact associated with increased levels of biomarkers of complement activation, such as sC5b-9, C3a, and C5a [42]. The latter were demonstrated to be less efficient regulatory elements to inhibit the activation of the alternative complement pathway. A specific interaction between C3b and the A2 domain of VWFs was demonstrated [45]. These findings are in line with published data showing high-affinity binding between VWF and C3b in surface plasmon resonance experiments and colocalization of C3/C3b with ULVWF on histamine-stimulated HUVECs [46,47]. Upon a trigger event that activates or injures the endothelial cells, ULVWF multimers are secreted from Weibel–Palade bodies on the endothelial cells’ surfaces, and the binding of C3b may amplify the alternative complement pathway cascade by forming a C3 convertase complex. In normal subjects, ADAMTS13 cleaves the ULVWF multimers from the cell surface and prevents the activation of the alternative complement pathway, maintaining homeostasis. Normal VWF multimers act as cofactors for complement factor I, an inhibitor of complement activation via the cleavage of complement C3b [48]. Zheng and colleagues demonstrated in a murine model that ADAMTS13 deficiency and the dysregulated complement pathway have synergistic effects [49]. KO mice with *Adamts13* (*Adamts13*^−/−^) or loss-of-function heterozygous mutant complement factor H (cfh) mutations (*cfh*^W/R^) did not develop spontaneous TTP. By contrast, animals with both *Adamts13*^−/−^ and *cfh*^W/R^ developed a TTP. Of note, the homozygous *cfh*^R/R^ form only developed a TTP independently from *Adamts13*^−/−^ [49]. The interplay between ADAMTS13 activity and complement activation was also shown in human iTTPs, where the mortality rate correlates with complement dysregulation [43]. How complement dysregulation during the acute phase of disease could provide better prognostic elements concerning disease recrudescence, relapse, and mortality predictions remains to be established.

## 4. Clinical Symptoms of First Episodes and Relapse Incidence of iTTP

The classical symptoms of a first episode of iTTP are represented by variable neurological symptoms (from headache to seizures and coma), severe thrombocytopenia, MAHA with schistocytes, and different degrees of multiorgan failure (heart, kidney, gastro-intestinal system) [8]. TTP is a rare disease that mainly affects young people and requires urgent treatment. Despite adequate treatment, 10% of patients will die from this disease, and up to 50% of patients will have recurrent episodes [28]. A recent study was performed in Spain with the application of the French TMA Reference Center Score and the mortality in TTP Score in 20 patients suffering from de novo and relapsed episodes of iTTP [50]. The median age of these patients was 46 (IQR 39–56). Of interest, among exacerbation and relapse episodes, thirteen (45%) were relapses of a previously diagnosed TTP, 14% corresponded to second episodes, 14% to third episodes, 7% to fourth episodes, and 10% to fifth episodes or beyond. The median time elapsed from the previous episode to relapse was ≈36 months (IQR 9–82 months). Thirteen episodes (45%) were associated with potential triggers. The most frequent triggers were infections/antibiotic use (52%), surgery (16%), the onset of an autoimmune disease (16%), pregnancy (8%), and cocaine use (8%). A real-world analysis of a large US health records database found high mortality and morbidity in patients with iTTP, despite treatments with plasma exchange and immunosuppression [51]. The relapse rate observed in this study was 11% over a shorter follow-up period of 4 years [51], whereas the exacerbations (within 1 month since the diagnosis and onset of therapy) were equal to 17% [51]. The observed mortality rate of 14% among patients with one or more iTTP episodes is consistent with the 8–20% reported in the literature for patients treated with plasma exchange and immunosuppression [51].

Hence, from these findings, a high incidence of exacerbation and relapse episodes emerged from these real-world data for iTTP patients. Thus, once iTTP is triggered, the prevalence and incidence of disease relapse are significantly higher than analog phenomena in hereditary TTP (see below). These observations deserve adequate hypotheses about possible differences in the pathophysiology of the two forms, which share the same ADAMTS13 deficiency.

## 5. Clinical Symptoms of First Episodes and Relapse Incidence of Hereditary TTP

Hereditary TTP is considered a rare syndrome, as most estimates suggest an overall prevalence of <1/1 × 10^6^. However, a greater prevalence has been observed in the Central Norway Health Region, where the estimated prevalence of the p.R1060W mutation is 16.7 cases per million people [52]. More than 200 ADAMTS13 mutations have been identified in all of the ADAMTS13 protein domains [53,54,55]. Of note, some missense ADAMTS13 single-nucleotide polymorphisms have also been identified, which, in some cases, are in strong linkage disequilibrium with specific ADAMTS13 mutations, influencing their molecular effects [56,57,58]. The clinical manifestations of hereditary TTP (also referred to as the Upshaw–Schulman syndrome) are typical of other TMA forms and comprise thrombocytopenia, MAHA, and multiorgan failure. Although patients with hereditary TTP are at increased risk for typical manifestations of microvascular thrombosis throughout their lives, two periods appear to be associated with high risks. These periods are represented by neonatal life and pregnancy/puerperium. In the former case, characterized by jaundice, anemia, and severe thrombocytopenia, the syndrome may also be fatal and diagnosed only post-mortem, as reported [59]. Beside pregnancy/puerperium and neonatal life, other clinical settings, such as infections and alcohol abuse, may be characterized by hereditary TTP episodes. In a recent study based on data from 87 patients followed in the Hereditary TTP Registry (clinicaltrials.gov #NCT01257269), a wide variety of incidence and severity of clinical manifestations of this syndrome have been reported [60]. Hereditary TTP exacerbations can mimic iTTP but may be less acute in onset, and renal failure is more common. The laboratory parameters may only be slightly perturbed or even normal. It is possible that cTTP patients can present neurologic symptoms with essentially normal platelet counts. Likewise, one of the most frequent symptoms is headache, which may occur without significant thrombocytopenia or any organ failure. It is not uncommon that cTTP patients, even with ADAMTS13 levels of 1–3%, are completely asymptomatic. Globally, the data provided by the above registry showed that the annual incidence of acute episodes is equal to 0.41 (95% CI, 0.30–0.56) for patients without regular plasma treatments. Moreover, an annual low incidence rate of acute episodes of ≤0.5 was recorded in 67.3% of patients with an ADAMTS13 activity of <1% [60]. Notably, many patients that are homozygous for the c.4143_4144drupA mutation have an ADAMTS13 activity of <1% but widely varying clinical courses [61]. Based on the above findings, an interesting question may emerge, which is as follows: Why is the comparably severe deficiency of ADAMTS13 observed in both iTTP and cTTP associated with a much higher incidence of exacerbations and relapse episodes in the former? In the next section, we will discuss the potential reason for this apparent discrepancy, remarking on the possible direct involvement of the anti-ADAMTS13 antibodies on the severity and prevalence of thrombotic complications in iTTP.

## 6. The Role of Anti-ADAMTS13 Antibodies in the Pathological Complications of iTTP

All of the IgG subclasses of anti-ADAMTS13 antibodies were detected in patients with iTTP, with the IgG(4) isotype followed by IgG(1) and IgA antibodies dominating the anti-ADAMTS13 immune response [62]. IgG(1) seems to be the dominant subclass during the first acute episode, whereas IgG(4) would be dominant during or following a relapse [63]. The IgG(1) subclass is a potent inducer of inflammation, as it can effectively bind to Fcγ receptors and activate the classical pathway of the complement system. At variance, IgG(4) tends to be anti-inflammatory, as it is not able to activate the complement system via the classical pathway and binds to Fcγ receptors with low affinity [64]. Hence, the levels of IgG(4) could be efficiently monitored for the identification of patients at risk of disease relapse. In a recent and elegant study, anti-ADAMTS13 IgG and their F(ab)’2 fragments, purified from 62 iTTP patients but not free from heme and nucleosomes, showed a specific effect on endothelial cells (ECs), in which the autoantibodies elicited in vitro the Ca^2+^-mediated activation of endothelial cells [65]. However, it should be noted that some authors found that free heme and nucleosomes may induce degranulation of WPBs through TLR4 ligation [66]. Likewise, free heme can facilitate the activation of the complement pathway on the surface of endothelial cells, empowering the dysregulation of this compartment and rendering it prone to thrombotic phenomena [67,68]. Plasma from TTP patients was demonstrated to induce endothelial cell apoptosis and platelet activation [69]. The possible involvement of endothelial cell dysregulation in the thrombotic phenomena of TTP is a debated topic. It is still unclear whether the endothelial activation detected through measurements of endothelial biomarkers is the cause or a consequence of the disease. Increased levels of endothelial microvesicles in TTP patients during the acute phase of the disease were previously documented, whereas during the remission period, the endothelial microparticles strongly decreased [70]. Of interest, TTP plasmas induce procoagulant endothelial microvesicle generation from cultured brain and renal microvascular endothelium [70]. Likewise, an elevated level of circulating endothelial cells was described in a prospective multicentric study in France during the acute phase of TTP, which was normalized during remission [71]. However, further studies are needed to validate in vivo the hypothesis concerning the activation of endothelial cells by anti-ADAMTS13 antibodies. It should be noted, however, that the activation and possible apoptosis of endothelial cells by the purified anti-ADAMTS13 antibodies in vitro caused a rapid VWF release and P-selectin exposure on human dermal microvascular endothelial cell (HMVEC-d) surfaces, associated with angiopoietin-2 and endothelin-1 secretions from the Weibel–Palade bodies [72]. Notably, calcium (Ca^2+^) blockades with the calcium chelator MAPTAM (1,2-bis-5-methylaminophenoxylethane-NNN’-tetraacetoxymethyl acetate) significantly decreased the VWF release [72]. The authors of this study did not report the molecular mechanisms through which the anti-ADAMTS13 antibodies can induce Ca^2+^ liberation inside endothelial cells. Ca^2+^ signaling in ECs plays a key role in the release of several biochemical mediators, such as NO, prostacyclin (PGI_2_), platelet activating factor (PAF), VWF, tissue plasminogen activator (tPA), and tissue factor pathway inhibitor (TFPI). Ca^2+^ signaling in ECs involves an initial increase in the intracellular free [Ca^2+^] ([Ca^2+^]_i_). The rise in [Ca^2+^]_i_ takes place via second messenger-mediated processes, which, in turn, trigger the release from intracellular Ca^2+^ stores in the endoplasmic reticulum (ER), and this is followed by Ca^2+^ entry from the extracellular space. This mechanism of Ca^2+^ entry can involve ER Ca^2+^ depletion but also directly receptor-activated Ca^2+^ entry. Of interest, the influx of Ca^2+^ into ECs occurs via some channels that are not gated by voltage. Among these, several polymodal transient receptor potential cation/canonical channels (TRPCs) have been shown to mediate the endothelial Ca^2+^ influx in ECs [73]. Seven TRPC isoforms (TRPC1 to 7) have been described in mammalian species, which have been classified into the following four subfamilies on the basis of their structural homologies and functional similarities: TRPC1, TRPC2, TRPC4/5, and TRPC3/6/7 [74]. TRPC5 expression has been found in many cell types with inherited mechanosensitive Ca^2+^ influx, including ECs [75]. TRPC5 channels have been involved in different physiological and pathophysiological processes, including endothelial functions and vascular smooth muscles [75]. TRPC5 participates in endothelial cell injury and dysfunction, migration and proliferation of vascular smooth muscle cells, cardiac hypertrophy, and lipid deposition. Moreover, a pharmacological block of the TRPC5 channels can inhibit atherosclerotic plaque progression, improve renal function, play a synergistic role in improving the prognosis of CVD patients, and, importantly, prevent depression and anxiety [75], the latter being a common symptom of the long-term effects of iTTP [76]. Thus, TRPC inhibitors may represent an intriguing target for the pharmacological control of cardiovascular morbidity and mortality in iTTP. A vast class of TRPC inhibitors has been synthesized, comprising pyrazoles, 2-aminoquinolines (among these compounds, ML204 is a potent inhibitor of TRPC5), phenylethylimidazoles, piperazine/piperidine analogues, naphthalene sulfonamides, N-phenylanthranilic acid, polyphenols, and 2-aminothiazoles [65]. However, due to the tissue-specificity of TRPC expression, the possibility of obtaining a selective and positive effect on endothelial cells is still far from being reached. However, independently from this possible pharmacological intervention, the direct effect of anti-ADAMTS13 antibodies on endothelial cells cannot be ignored any longer in the pursuit of better therapeutic controls for the severe cardiovascular complications of iTTP.

## 7. Effects on Therapies of the Different Pathogenetic Effectors of cTTP and iTTP

The different pathogenetic effectors and varying clinical phenotypes described above in cTTP and iTTP determine the different therapeutic approaches for their treatments. Plasma infusion may be administered in both clinical forms, but it is predominantly used in cTTP while waiting for the final approval of recombinant ADAMTS13 by regulatory agencies [77]. By contrast, plasma exchange is mandatory for iTTP to eliminate the anti-ADAMTS13 antibodies and, at the same time, provide sufficient amounts of ADAMTS13. However, plasmapheresis may be administered, even to patients with relapses of the refractory forms of cTTP, to deliver sufficient amounts of ADAMTS13 and avoid volume overload. As for caplacizumab, this drug is approved for the treatment of iTTP and is given daily after plasmapheresis plus 30 days following remission. However, off-label use of caplacizumab has been previously reported in a cTTP case with severe multiorgan thrombotic microangiopathy and was associated with a positive outcome [78]. The substantial difference concerns the immunosuppressive therapy (high doses of corticosteroids, cyclophosphamide, rituximab, etc.) that is administered in iTTP only, while theoretically, in severe cTTP cases with complete ADAMTS13 deficiency, the formation of inhibitory antibodies against the metalloprotease could derive from the treatment with plasma.

## 8. Conclusions

**Figure 1:**
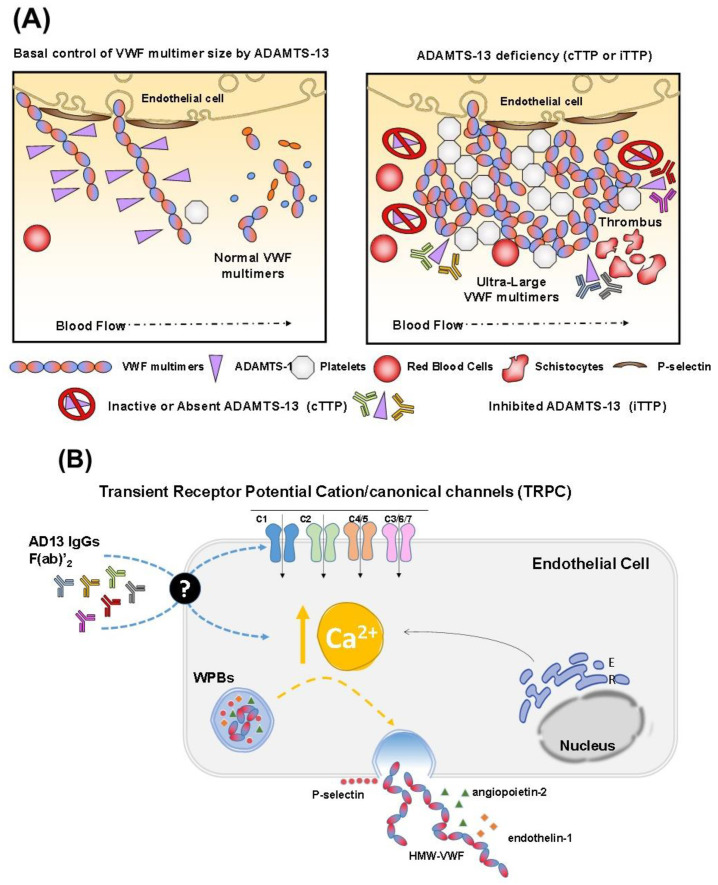
Scheme of the canonical (**A**) and putative (**B**) mechanisms responsible for the thrombotic phenomena in iTTP and cTTP, occurring at the endothelial level. In the putative mechanism shown in (**B**), endothelial activation occurs through stimulation of the Ca^2+^ entry and signaling responsible for the secretion of Weibel–Palade bodies and other biochemical mediators able to activate platelets.

In conclusion, the understanding of the immunological basis of iTTP, which accounts for the majority of TTP cases, has progressively increased in the last few years. Plenty of previous studies investigated the contribution of antibodies against different ADAMTS13 domains to the inhibitory potential in plasma and revealed how these autoantibodies may cause both accelerated clearance with depletion of the metalloprotease and inhibition of its activity (Figure 1A). However, recent studies have also noted a direct activity of the various IgG subclasses on the activation of endothelial cells and platelets responsible for the pathological phenomena of iTTP (Figure 1B). Hence, the application in the future of more specific drugs able to control even the direct cellular effects of the TTP-associated autoantibodies, besides the immunological response, will provide an additional strategy for disease control, together with the fundamental therapeutic tools represented by plasma exchange, immunosuppressors, and caplacizumab.

## Figures and Tables

**Table 1:** Diagnostic features of different thrombotic microangiopathies (TMAs).

Clinical Form	Diagnostic Features
Thrombotic Thrombocytopenic Purpura (TTP)	ADAMTS13 deficiency
Infection-associated TMA	Shiga-toxin, Streptococcus pneumonia, Campylobacter jejuni, Cytomegalovirus, Human immunodeficiency virus, Parvovirus B19, Epstein–Barr virus, BK virus, Influenza
Complement-mediated hemolytic uremic syndrome (HUS)	Dysregulation of complement factors and their inhibitors
Secondary TMAs	Cancer, Transplantation, Antiphospholipid antibody Systemic lupus erythematosus syndrome, Scleroderma, Vasculitis/ glomerulonephritis
Disseminated intravascular coagulation	Sepsis, cancer
Drug-induced TMA	Calcineurin or mTOR inhibitors, Quinine, Interferon Vascular endothelial growth factor or proteasome inhibitors Estrogen/progesterone, Gemcitabine/ mitomycin C, Cocaine
Malignant hypertension-induced TMA	Extreme levels of blood pressure, severe headache, papilledema
Pregnancy-associated TMA	HELLP (hemolysis, elevated liver enzymes, and low platelets) syndrome, HUS, TTP
Metabolism-associated TMA	Cobalamin responsive methylmalonic acidemia, mutation of Diacylglycerolkinase epsilon
COVID-19 associated TMA	SARS-COV2 infection, evidence of microangiopathy
